# Reproductive health problems in rural South African young women: risk behaviour and risk factors

**DOI:** 10.1186/s12978-018-0581-9

**Published:** 2018-08-15

**Authors:** Hashini Nilushika Galappaththi-Arachchige, Siphosenkosi G. Zulu, Elisabeth Kleppa, Kristine Lillebo, Erik Qvigstad, Patricia Ndhlovu, Birgitte Jyding Vennervald, Svein Gunnar Gundersen, Eyrun Floerecke Kjetland, Myra Taylor

**Affiliations:** 1 Norwegian Centre for Imported and Tropical Diseases, Department of Infectious Diseases Ullevaal, Oslo University Hospital, Postboks 4956 Nydalen, 0424 Oslo, Norway; 2 Institute of Clinical Medicine, University of Oslo, Oslo, Norway; 3 Department of Infection Prevention and Control, Nelson R Mandela School of Medicine, College of Health Sciences, University of KwaZulu-Natal, Durban, South Africa; 4 Department of Gynaecology, Women and Children’s Division, Ullevaal University Hospital, Oslo, Norway; 5 Imperial College London, Hammersmith Campus, London, UK; 6 Section for Parasitology and Aquatic Pathobiology, Faculty of Health and Medical Sciences, University of Copenhagen, Copenhagen, Denmark; 7 Research Unit, Sorlandet Hospital, Kristiansand, Norway; 8 Department of Global Development and Planning, University of Agder, Kristiansand, Norway; 9 Discipline of Public Health Medicine, Nelson R Mandela School of Medicine, College of Health Sciences, University of KwaZulu-Natal, Durban, South Africa

## Abstract

**Background:**

South African young women continue to be vulnerable, with high prevalence of teenage pregnancy, HIV, sexually transmitted infections (STIs) and female genital schistosomiasis (FGS). This study seeks to examine the underlying factors that may be associated with these four adverse reproductive health outcomes.

**Methods:**

In a cross-sectional study of 1413 sexually active of young women, we explored these four adverse reproductive health outcomes by considering socio-demographic factors, socio-economic factors, sexual risk behaviour, substance abuse and knowledge about reproductive health by using a questionnaire. Consenting participants were asked about previous pregnancies and were tested for HIV, STIs and FGS. Multivariable regression analyses were used to explore the factors associated with these four reproductive health outcomes.

**Results:**

1. Early pregnancy: Among the young women, 44.4% had already been pregnant at least once. Associated factors were hormonal contraceptives, (adjusted odds ratio (AOR): 17.94, 95% confidence interval (CI): 12.73–25.29), and sexual debut < 16 years (AOR: 3.83, 95% CI: 2.68–5.47). Living with both parents (AOR 0.37, 95% CI: 0.25–0.57) and having a steady partner (AOR: 0.43, 95% CI: 0.24–0.76) were identified as protective factors against pregnancy.
2. HIV: HIV prevalence was 17.1%. The odds of having HIV were higher in intergenerational (AOR: 2.06, 95% CI: 1.05–4.06) and intragenerational relationships (AOR: 1.51 95% CI: 1.06–2.15), compared to age-homogenous relationships. Other associated factors were: condom use (AOR: 1.60, 95% CI: 1.16–2.20), number of times treated for an STI (AOR: 1.32, 95% CI: 1.02–1.71), and total number of partners (AOR: 1.14, 95% CI: 1.03–1.28).
3. STIs: Participants who had at least one STI (40.5%) were associated with total partner number (AOR 1.17, 95% CI: 1.06–1.30), and testing HIV positive (AOR: 1.88, 95% CI 1.41–2.50).
4. FGS: FGS prevalence (19.7%) was associated with previous anti-schistosomal treatment (AOR: 2.18, 95% CI: 1.57–3.05).

**Conclusion:**

There is a high prevalence of pregnancy, HIV, STIs and FGS among sexually active young women in rural KwaZulu-Natal. Multidisciplinary approaches are urgently needed for educational and health literacy programs prior to sexual debut, and health care facilities, which should be made accessible for young women.

## Plain English summary

School-attending young women (16–20 years) from KwaZulu-Natal, South Africa are highly affected by adverse reproductive health outcomes such as teenage pregnancy, HIV, sexually transmitted infections (STIs), and female genital schistosomiasis (FGS). Unlike the first three reproductive outcomes, FGS is caused by a fresh-water parasite that may cause discharge and sores in the genital tract in women. Affected women may present with lower abdominal pain or infertility. In this study we explore the teenage social and behavioural factors that are associated with these four adverse reproductive health outcomes. Early sexual debut, multiple partners, having an older partner, not living with both biological parents, and having previously received treatment for schistosomiasis were associated with adverse reproductive health outcomes. Implementing educational and health literacy programs, in addition to making health care facilities accessible may improve the health of young women.

## Background

School-attending young women from KwaZulu-Natal (KZN), one of South Africa’s poorest provinces, are markedly affected by reproductive health outcomes such as teenage pregnancy, HIV, sexually transmitted infections (STIs), and female genital schistosomiasis (FGS) [1–5].

In KZN, pregnancy among school-attending young women is very common, and about one third of young women have had a child by the age of 20 [1, 5]. This impacts on the mothers’ schooling, and subsequent employment and earnings [6]. In turn, this also affects the child, who is born into poverty [6].

Worldwide, approximately 36.7 million are living with HIV and South Africa has the highest HIV prevalence in the world, with approximately 18% (7 million) of the global burden of HIV [7]. Young women aged 15–24 years are at particular risk of HIV and accounted for 20% of new HIV infections globally in 2015 and most of these are in Sub-Saharan Africa [8]. KZN has the highest HIV burden in South Africa with an estimated HIV prevalence of 44.4% reported in the antenatal survey of 2015 [9]. HIV prevalence in young women (15–24 years) is approximately four times that of men in the same age group, furthermore, in South Africa, young women acquire HIV at a much younger age than their male counterparts [10].

STIs, particularly *Chlamydia trachomatis*, *Neisseria gonorrhea*, *Trichomonas vaginalis* and syphilis, continue to be endemic in KZN, especially among sexually active young women [3, 11]. Many STI infections are asymptomatic [11]. Consequently, many do not seek care, and therefore remain untreated [12]. Untreated STIs may cause pelvic inflammatory disease (PID), increase the risk of ectopic pregnancies and cause infertility [13].

In *Schistosoma (S.) haematobium* endemic areas such as KZN, another neglected cause of reproductive morbidity in young women is female genital schistosomiasis [2, 14]. FGS is not caused by sexual intercourse, it is acquired through contact with contaminated fresh-water and can cause lesions in the reproductive tract [15]. Affected women may however suffer from symptoms similar to those of STIs (e.g. abnormal discharge) and can often be misdiagnosed [15, 16]. In addition, studies have shown that women with *S. haematobium* infection have 2–4 times increased odds of having HIV [17, 18].

Over the past decades, studies have shown that adverse reproductive health outcomes are largely due to a number of social and behavioural factors [19–24]. Moreover, relationship issues have also been found to contribute to risks for young women, such as the age difference between sexual partners (with an older male partner), multiple partners, and HIV positive partners [25]. In the absence of parental care and financial stability, young women may be more prone to involve themselves in transactional sex [26]. FGS is also multifactorial, with socio-economic factors playing a large role and lack of access to treatment (praziquantel) [2, 15, 27].

In this study we examine four common adverse reproductive health outcomes, (1) teenage pregnancy, (2) HIV, (3) STIs and (4) FGS, in sexually active young women in KZN. We explored these four outcomes by considering socio-demographic factors, socio-economic factors, sexual risk behaviour, substance abuse and knowledge about reproductive health. Furthermore, due to the similar clinical picture that FGS shares with STIs, we explored if there were any associations between the participants’ knowledge about STIs and FGS. By understanding the underlying factors associated with these four adverse reproductive health outcomes, we may be able to help prevent these outcomes through intervention programs in collaboration with policy makers, health care workers, and educationists.

## Methods

### Study area and recruitment

The young women were part of a cohort of high-school students included in a cross-sectional study on female genital schistosomiasis in rural KZN, South Africa. The study investigated school-attending young women because schools provide a useful access point for mass treatment for schistosomiasis, as recommended by the World Health Organization. The study was undertaken in high schools from three districts in KZN. The recruitment took place from 2011 to 2013. Targeting high-schools with more than 300 pupils, situated in rural areas in Ilembe, uThungulu and Ugu districts, we randomly selected 70 schools in schistosomiasis-endemic areas (below 400 m altitude for the study) [28]. The included schools were visited during the least busy part of the school year, in order to have a minimal effect on their studies. Dates for possible investigations were provided by the teachers and subsequently discussed with the young women individually. All students from grade eight and above (16 years and older) were invited to participate in the recruitment phase of the study, however, only those who had been sexually active were included in the study. In order to determine who were sexually active, without disclosing this as the inclusion criterion, we used a questionnaire that also had many other questions. Furthermore, parents were informed about the study and participants who provided written informed consent were included. We excluded those who were pregnant, older than 20 years and those who did not consent to have a gynaecological examination. We further excluded those who had inconsistencies in the reported sexual debut age, age of menarche, and age of first pregnancy (e.g. if someone reported pregnancy before sexual debut or menarche). We calculated that with a sample size of minimum 1400 participants, we would have statistical power of 80% or more for detecting differences between groups of interest when at least 250 of the participants had a risk factor.

### Questionnaire

A structured questionnaire, which was developed after reviewing the literature, was piloted among young school going women. The questionnaire was developed in English, translated into isiZulu (the local language) and translated back into English to ensure accuracy. It was used to interview all the consenting participants. Trained research assistants performed the interviews in their local language (isiZulu). The questionnaire contained questions designed to assess demographic, socioeconomic and reproductive health factors. We asked questions on household characteristics, participants’ relationship status, total number of lifetime partners, age of their oldest partner, sexual debut age and whether they had ever been pregnant. The questionnaire also enquired about contraceptive use (current hormonal and condom use), HIV and STIs related issues, such as: whether they had previously been tested for HIV, and whether they know what an STI is. We further asked about previous treatment history for both STIs and schistosomiasis. The age of alcohol debut and substance use was included as well.

### Clinical examination and laboratory analyses

Prior to the gynaecological examination, the investigating clinician explained in detail about the procedure to each participant and answered any questions related to the examination. The examination started with a visual inspection of the vulva, vagina and cervix, followed by a photocolposcopic examination using an Olympus OCS 500 Colposcope with a mounted Olympus E420 (10 Mpx) single lens reflex (SLR) camera or a Leisegang colposcope with a mounted Canon EOS 650D (18 Mpx) SLR camera [29]. Lesions in the lower reproductive tract were described as: sandy patches, homogenous yellow sandy patches, rubbery papules, abnormal blood vessels, genital ulcers, leukoplakia, tumors and rubor [16]. A positive FGS diagnosis would be given if they had one of the characteristic FGS lesions, such as sandy patches, homogenous yellow sandy patches or rubbery papules [30].

Cervico-vaginal lavage samples (CVLs) were collected by spraying 10 mL saline on the cervix four times, followed by drawing this back into the syringe [16]. The CVL was analysed for *Neisseria gonorrhoea* and *Chlamydia trachomatis* using a strand displacement assay (ProbeTec CT/GC, Becton, Dickinson and Company (BD), Franklin Lakes, NJ, USA) [20]. The CVL was also analysed using an in-house PCR (Laboratory of Infection, Prevention and Control, University of KwaZulu-Natal (UKZN), Durban, South Africa) detecting *Trichomonas vaginalis* [20]*.*

Thirty milliliters blood was collected in sterile acid-citrate-dextrose anti-coagulant Vacutainer tubes (BD) [19]. Using the South African Department of Health’s protocol, HIV testing was done using the Bioline Rapid test HIV (New Jersey, United States) and confirmed using Sensa TriLine HIV Test Kit (Pantech, Durban, South Africa). Syphilis screening was performed at Laboratory of Infection, Prevention and Control, UKZN, Durban, South Africa, using rapid plasma reagin (RPR, Macro Vue test 110/112, BD), and positive tests were confirmed using *Treponema pallidum* hemagglutination assay (TPHA, Omega Diagnostics Group PLC, Alva, Scotland, UK) [23]. All serology was done on serum samples that had been stored at − 80 °C.

### Statistical analysis

Teenage pregnancy was defined as having had at least one pregnancy (live birth, ectopic pregnancy, spontaneous abortion, or termination of pregnancy) before the age of 20. The STI-variable would be coded positive if the participant had tested positive for one or more STIs (*T. pallidum, T. vaginalis, N. gonorrhoea or C. trachomatis*). Further, we calculated the number of STIs for each of the participants, excluding participants who had any missing STI-results. To assess the risk entailed by having an older partner, we calculated the age difference between the young woman’s current age and the age of the oldest partner. Then the age difference was further divided into age-homogenous (≤ 4 years age difference), intragenerational (5–9) and intergenerational relationships (≥10), as defined in the literature [31]. Level of education was categorised as secondary (grade 8–12) or tertiary education.

Statistical analyses were done using Statistical Package for Social Sciences (SPSS) version 24 (IBM, Chicago, IL, USA). Due to the binary coding of the outcome variables, we used logistic regression (nonparametric) to evaluate the association between each of the four outcome variables and social and behavioural factors. We calculated odds ratio (OR) and the corresponding 95% confidence intervals (CI). Associations were considered statistically significant within a significance level of 0.05. This significance level was also used for inclusion of risk behaviour and social and behavioural factors in the multivariable logistic regression analyses. Due to a strong age-dependence, alcohol debut age was stratified for current age.

We created Venn diagrams using Venny version 2.1 (Juan Carlos Oliveros, http://bioinfogp.cnb.csic.es/tools/venny/) to evaluate the co-occurrence of adverse reproductive health outcomes [32]. We further assessed the social and behavioural factors associated with the two and three most common co-occurring reproductive health outcomes.

## Results

### General characteristics of the study population

**Fig. 1:**
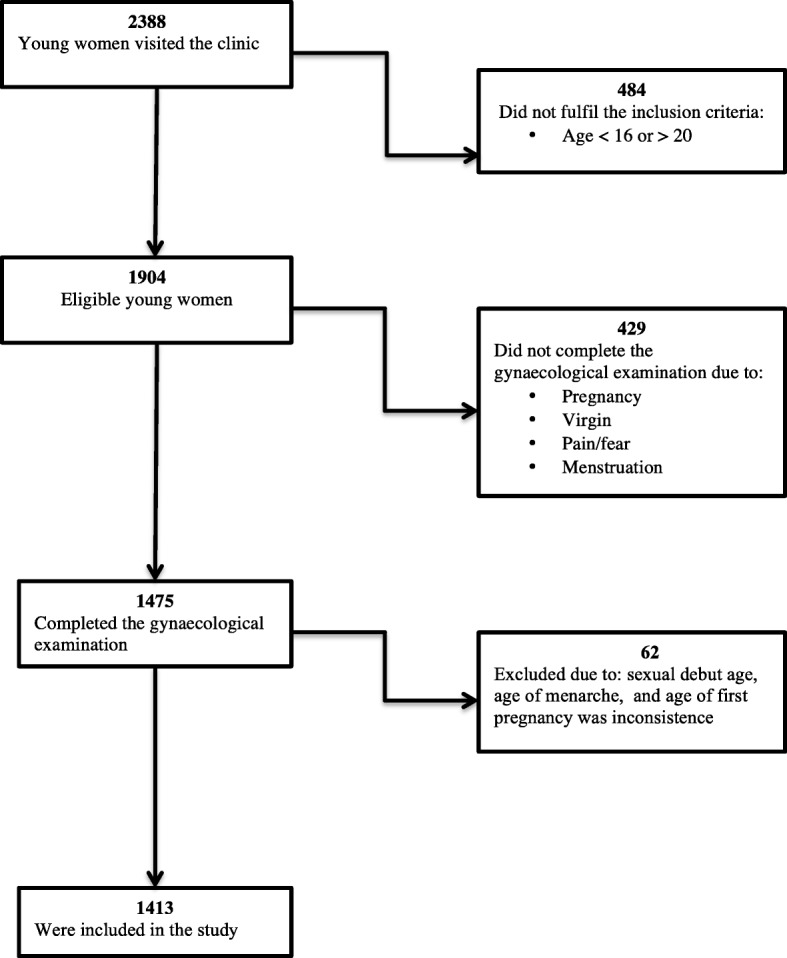
Flowchart showing participant selection procedure

In total, 1413 sexually active young women of median 18 years (range 16–20) were included in the study. Figure 1 shows the participant selection procedure. The characteristics of the participants’ households are presented in Table 1.

**Table 1:** Household structure, educational and socio-economic characteristics of the study participants

	Proportion of participants
Household structure	
Median number of people in the household (range)	6 (1–18)
Median number of adults (≥18 years) in the household (range)	4 (0–14)
Median number of children in the household (range)	2 (0–11)
Living with both of her biological parents	19.8% (279/1411)
Living with one of the biological parents or other adults	80.0% (1129/1411)
Did not have any adults in the household	0.2% (3/1411)
Participants 18 years or older were the only adult in the household	1.3% (19/1411)
Highest level of education in the household (excluding study participant)	
High School	88.0% (1236/1404)
Tertiary level of education	12.0% (168/1404)
Proportion of adults employed in the household	
No adults employed	43.1% (607/1408)
1–49% of adults employed	30.8% (424/1408)
≥ 50% of adults employed	26.1% (367/1408)

The median age of menarche was 14 years (range 8–19) and the median age of first sexual debut was 16 years (range 10–20). At the time of the interview, 94.7% (1333/1407) of the young women reported having a steady partner and reported a median of two lifetime partners (range 1–22). The median age difference between the participants and their oldest partner was 3 years (range: less than 5 years and up to 40 years). The majority of the young women reported to have age-homogenous relationships (68.9% 966/1403), followed by intragenerational relationship (26.8% 376/1403) and intergenerational relationships (4.3% 61/1403). Only 2.1% (30/1408) reported receiving money or gifts for sexual favours and 7.5% (106/1409) reported having been sexually abused at some point in their lives. Less than half reported to have used a condom during their last sexual intercourse (40.0%, 494/1234). Hormonal contraceptives were used by 29.7% (418/1409) at the time of the study and of these, an injectable contraceptive was the most common contraceptive method (96.7%, 404/418).

The median age for alcohol debut was 16 years (range 10–20). More than half, 59.0% (834/1413), reported to have tried alcohol. Illicit drugs were used by 3.8% (54/1411) of these young women, and 18.3% (258/1406) reported that their friends were using illicit drugs. The most common drug reported to be used by the participants was inhalation of benzine (4.3%, 61/1411), followed by smoking of cannabis (2.6%, 36/1410), inhalation of glue (1.1%, 15/1410) and taking ecstasy pills 0.6% (8/1410). Only one person reported use of injectable drugs.

### Social and behaviour factors associated with teenage pregnancy

As many as 44.4% (627/1413) of the school-attending, sexually active young women reported having been pregnant at least once in their lifetime. One inclusion criterion for the study was admitting to being sexually active. Only 2.8% (39/1413) reported having been pregnant twice and one single participant reported having been pregnant three times. Miscarriage was reported by 1.3% (8/627), stillbirth by 1.6% (10/627) and 2.6% (16/627) reported that the child died during childhood. The median age of first pregnancy was 17 years (range 13–20). Social and behavioural factors associated with pregnancy are presented in Table 2. The odds of a participant having been pregnant were almost four-fold higher (*p* < 0.001) in those who reported sexual debut before 16 years of age. Current contraceptive use was more common among those who had been pregnant (*p* < 0.001). Those living with both parents had a reduced risk of being pregnant (*p* < 0.001) as did those who had a steady partner (*p* = 0.005).

**Table 2:** Logistic regression analysis of STIs, social and behavioural factors associated with pregnancy

Social and behavioural factors	Odds Ratio (95% CI)	Adjusted Odds Ratio (95% CI) ^a^
Age in years	1.76 (1.60–1.95)^**^	2.10 (1.84–2.39)^**^
Living with both parents	0.57 (0.42–0.76)^**^	0.39 (0.26–0.59)^**^
Level of education in the household ^b^	0.90 (0.66–1.23)	
Percentage of adults employed in the household	0.95 (0.86–1.04)	
Sexual debut before 16 years of age	1.56 (1.22–1.98)^**^	3.64 (2.56–5.17) ^**^
Ever been sexually abused	1.17 (0.79–1.74)	
Ever been paid to have sex	1.65 (0.79–3.42)	
Number of lifetime sexual partners	0.97 (0.89–1.05)	
Have a steady partner now	0.56 (0.35–0.90)^*^	0.44 (0.25–0.78)^*^
Age difference to oldest sexual partner	1.01 (0.98–1.04)	
Age difference to older partner (years)		
Age-homogenous (0–4)	1	
Intragenerational (5–9)	1.09 (0.86–1.38)	
Intergenerational (≥ 10)	1.64 (0.98–2.77)	
Currently using hormonal contraceptive	16.18 (11.9–22.00)^**^	18.28 (12.99–25.74) ^**^
Know what an STI is	1.23 (1.00–1.62)	
Tested positive for an STI at our clinic^c^	1.01 (0.82–1.26)	
Tested positive for HIV at our clinic	1.26 (0.95–1.66)	
Taking illicit drugs	1.00 (0.58–1.73)	
Alcohol debut age (stratified by current age)		
Current age 16	1.14 (0.67–1.94)	
Current age 17	0.95 (0.75–1.20)	
Current age 18	0.92 (0.77–1.11)	
Current age 19	0.90 (0.75–1.10)	
Current age 20	0.86 (0.70–1.06)	

*CI* confidence interval

^*^p < 0.05; ^**^p < 0.001

^a^All variables in the table with *p* < 0.05 in univariate analysis were included in the multivariable analyses

^b^Categorised as secondary (grade 8–12) or tertiary

^c^Tested for *C. trachomatis, T. vaginalis, N. gonorrhoea* and *T. pallidum*

### Social and behaviour factors associated with HIV

We found that 17.1% (241/1351) of the young women tested positive for HIV infection and 74.1% (181/241) did not know that they were HIV positive. Social and behavioural factors associated with having HIV infection are presented in Table 3. HIV seropositivity was associated with reporting use of a condom during the last sexual intercourse (*p* = 0.004). However, among those who knew they were HIV positive, we did not see a significant association with condom use during last sexual intercourse (*p* = 0.685). The odds of HIV increased depending on their current age (*p* = 0.006), their number of total lifetime partners (*p* = 0.008), and the number of times that they had been treated for an STI (*p* = 0.038). Furthermore, the odds of having HIV were higher in intragenerational and intergenerational relationships than in homogenous relationships (*p* = 0.022 and *p* = 0.039, respectively).

**Table 3:** Logistic regression analysis of STIs, social and behavioural factors associated with HIV

Social and behavioural factors	Odds Ratio (95% CI)	Adjusted Odds Ratio (95% CI)^a^
Age in years	1.31 (1.16–1.47)^**^	1.21 (1.06–1.40)^*^
Living with both parents	0.55 (0.36–0.86)^*^	0.65 (0.40–1.10)
Level of education in the household ^b^	0.71 (0.45–1.11)	
Percentage of adults employed in the household	0.96 (0.85–1.09)	
Sexual debut before 16 years of age	0.97 (0.87–1.07)	
Number of lifetime sexual partners	1.27 (1.13–1.42)^**^	1.17 (1.04–1.31)^*^
Have a steady partner	1.13 (0.58–2.18)	
Age difference to older partner (years)		
Age-homogenous (≤ 4)	1	1
Intragenerational (5–9)	1.58 (1.16–2.15)^*^	1.51 (1.06–2.15)^*^
Intergenerational (≥ 10)	2.46 (1.37–4.40)^*^	2.06 (1.05–4.06)^*^
Ever been sexually abused	1.55 (0.96–2.49)	
Ever been paid to have sex	2.12 (0.96–4.70)	
Condom used during last sexual intercourse	1.50 (1.11–2.04)^*^	1.60 (1.16–2.20)^*^
Using hormonal contraceptive	0.84 (0.62–1.15)	
Know what an STI is	1.24 (0.89–1.71)	
Number of times treated for an STI	1.60 (1.27–2.01)^**^	1.32 (1.02–1.71)^*^
Taking illicit drugs	1.99 (1.07–3.69)^*^	1.43 (0.65–3.11)
Alcohol debut age		
16	2.32 (0.92–5.80)	
17	1.27 (0.93–1.70)	
18	1.07 (0.81–1.40)	
19	1.23 (0.97–1.56)	
20	1.08 (0.86–1.35)	

*CI* confidence interval

^*^p < 0.05; ^**^p < 0.001

^a^All variables in the table with *p* < 0.05 in univariate analysis were included in the multivariable analyses

^b^Categorised as secondary (grade 8–12) or tertiary

### Social and behaviour factors associated with sexually transmitted infections

*Chlamydia trachomatis* was the most common STI with a prevalence of 24.8% (328/1325), followed by *Trichomonas vaginalis* at 17.9% (245/1371) and *Neisseria gonorrhoea* at 10.9% (145/1325). *Treponema pallidum* was the least common STI in this study group with a prevalence of 1.8% (24/1350). As many as 40.5% of the young women had one or more STIs. The numbers of concurrent STIs are presented in Tables 4, and 71.0% (991/1396) of the young women were aware of what an STI is. Few reported ever having received treatment for an STI (11.5%, 157/1360).

**Table 4:** Number of concurrent sexually transmitted infections

Number of STIs^a^	Participants (%)^b^
No STIs	719/1259 (57.1)
Single STI	401/1259 (31.9)
Two concurrent STIs	115/1259 (9.1)
Three concurrent STIs	23/1259 (1.8)
Four concurrent STIs	1/1259 (0.1)

^a^Tested for *C. trachomatis, T. vaginalis, N. gonorrhoea* and *T. pallidum*

^b^Participants with any missing lab results were excluded

In a multivariable regression model, using positive test result for any of the four STIs (*C. trachomatis, T. vaginalis, N. gonorrhoea* and *T. pallidum)* as the outcome variable, we found that testing positive for HIV was significantly associated with testing positive for an STI (AOR: 1.88, 95% CI: 1.41–2.50, *p* < 0.001). Furthermore, we found that the total number of sexual partners was significantly associated with having an STI (1.17, 95% CI: 1.06–1.30, *p* = 0.002). None of the other social and behavioural factors were found to be associated with having an STI and therefore these data are not reported.

### Social and behavioural factors associated with female genital schistosomiasis

We identified sandy patches in 19.7% (279/1413) of the young women and 18.7% (264/1410) reported having received anti-schistosomal treatment at some point in their life, whereas 35.2% (496/1410) could not recall whether they had received such treatment. In the multivariable analysis, controlling for the employment rate in the household, the only factor that remained associated with having FGS was previous anti-schistosomal treatment (AOR: 2.18, 95% CI: 1.57–3.05, *p* < 0.001). None of the other social and behavioural factors were found to be associated with having FGS and therefore these data are not reported.

### Co-occurrence of reproductive adverse outcomes

**Fig. 2:**
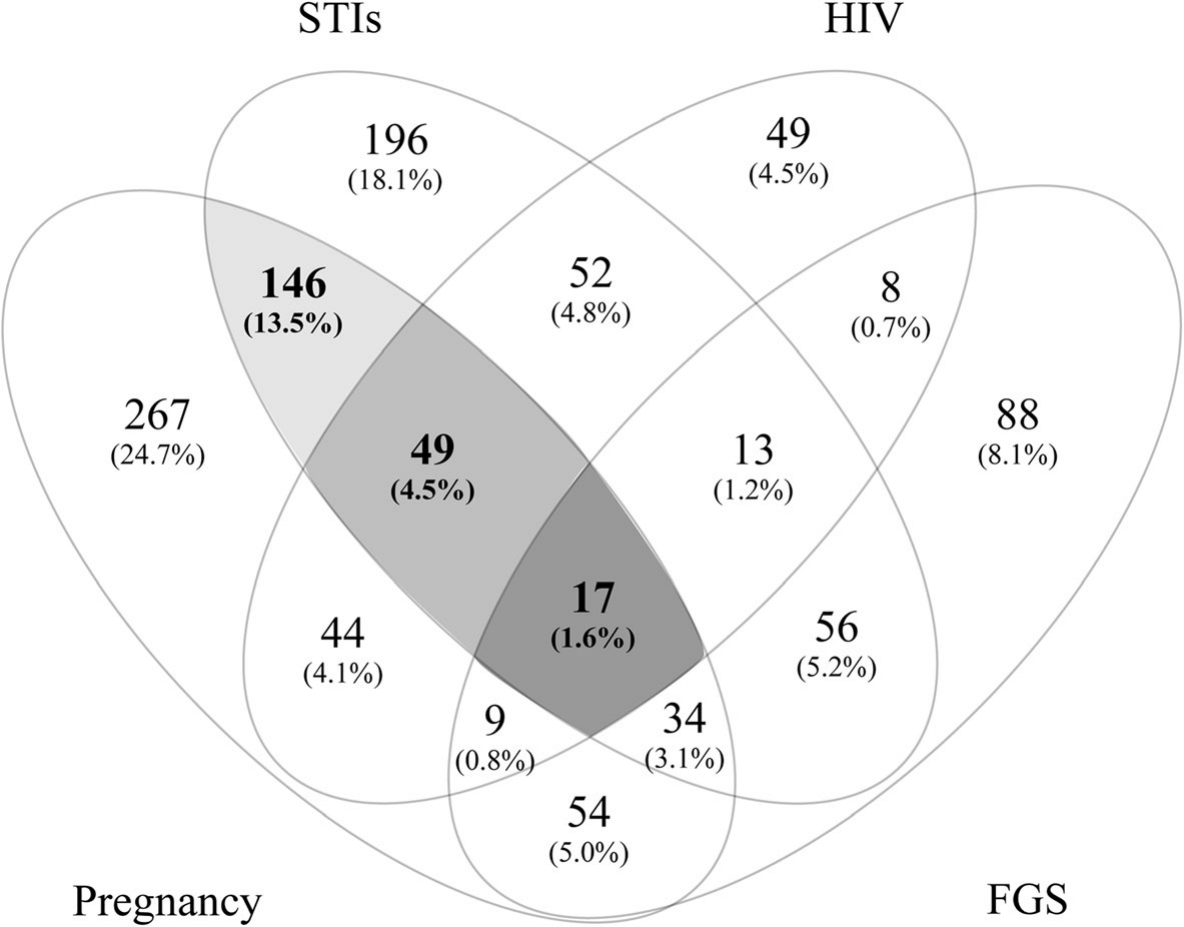
Venn diagram showing the number of participants having co-ocurring adverse reproductive health outcomes. STI = Sexually transmitted infections (*C. trachomatis, T. vaginalis, N. Gonorrhoea and T. pallidum*,) HIV = Human Immunodefiency virus, FGS = Female genital schistomiasis

As shown in Fig. 2, we found that 76.6% (1082/1413) had experienced an adverse reproductive health outcome (the sum of all cells in Fig. 2). Of these, 55.5% (600/1082) experienced a single outcome (the sum of all non-overlapping cells in Fig. 2), 33.3% (360/1082) experienced two outcomes (the sum of all the two-overlapping cells in Fig. 2), 9.7% (105/1082) experienced three outcomes (the sum of all the three-overlapping cells in Fig. 2) and 1.6% (17/1082) experienced four outcomes (the cell where all four outcomes overlap in Fig. 2, indicated by the darkest shade of grey). We were not able to do any further statistical sub-analyses in the latter group due to the small sample size.

The three most commonly co-occurring outcomes were pregnancy, STIs and HIV (the cell indicated by the medium shade of grey in Fig. 2, *n* = 49). Adjusting for age, hormonal contraceptive use and number of STI treatments in a multivariable regression analysis, we found that significant factors for experiencing these three reproductive health outcomes concurrently, were “thinks she has an STI now” (AOR 3.27, 95% CI: 1.20–8.92, *p* = 0.21) and total number of lifetime partners (AOR 1.35, 95% CI: 1.04–1.76, *p* = 0.026).

Of the participants who experienced only two adverse outcomes at the same time, we identified having been pregnant and having an STI to be the most commonly co-occurring outcomes (the cell indicated by the lightest shade of grey in Fig. 2, *n* = 146). In a multivariable regression analysis, we found that current use of hormonal contraceptive and age were significantly associated with having undergone teenage pregnancy and having a current STI (AOR: 3.74, 95% CI: 2.61–5.35, *p* < 0.001 and AOR: 1.20, 95% CI: 1.03–1.40, *p* = 0.021, respectively).

## Discussion

In this study, we explored four common adverse reproductive health outcomes, defined as (1) teenage pregnancy, (2) HIV, (3) sexually transmitted infections (STIs) and (4) female genital schistosomiasis (FGS), in sexually active young women in Kwazulu-Natal (KZN). To our knowledge, these outcomes have not been reported together before.

We found that living with biological parents was a strong protective factor against pregnancy, and may signify that parental supervision, monitoring and care are important factors that can reduce teenage risk behaviour [33, 34]. Use of hormonal contraceptives is an essential protective factor against unwanted pregnancies, however we found that those who were currently using hormonal contraceptives had higher odds of having been pregnant. This suggests that following the first pregnancy, the health care workers may have advised the use of hormonal contraceptives, and their parents may have further encouraged this [33–35]. This may also explain why our data showed low numbers of participants who reported being pregnant a second and third time. Unfortunately, overall contraceptive use among our participants remained low, which may indicate that teenagers do not seek or receive this service from the local clinics. It can be difficult to admit sexual activity to health care workers in the first years following sexual debut and this may result in missed opportunities for preventive care [36]. Among those who used contraceptives, an injectable contraceptive was the most common birth control method. Even though contraceptives are available free of charge in public health care settings, these women were living in rural areas and may neither have had a clinic nearby nor the funds to reach the nearest clinic [6]. Furthermore, young women who reported having a steady partner were less likely to have been pregnant. This could be because they had a stable relationship and were making shared decisions and exercising safer and healthier sexual practices [37].

Our study shows that most of the young women reported living with their biological mother, or with other adults although they were not their biological parents. Sadly, only less than one quarter reported living in a household with both biological parents. This may be a consequence of the HIV epidemic that has negatively affected the household structure in HIV endemic areas, where single or widowed females are the heads of the households [20]. It has also been reported that increasing numbers of children end up living with relatives rather than their biological parents or parents may have left their children with relatives in order to find work in the cities [20]. In our study, the presence of parents in the household reduced the odds of having HIV infection. However, when adjusted for other factors, this did not remain a protective factor for HIV. Similar to other studies, we found that having multiple partners and having an older partner increased the odds of testing positive for HIV [25, 38, 39]. A study from KZN found that a woman’s perceived risk of HlV infection from her partner was the most powerful predictor of condom use [40]. This may be reflected in our study as we see a significant association between condom use during last sexual intercourse and testing positive for HIV. However, we did not find an association between condom use and knowing whether they were HIV positive.

Confirming recent epidemiological studies from KZN, we found that the STI prevalence among young women remained high [3, 11]. In 2014, Naidoo et al. found that in KZN, women younger than 25 years, non-cohabitating and unmarried were at higher risk of getting an STI [3]. In agreement with previous studies, we found that young women who had multiple partners were more likely to be diagnosed with one or more STIs [25, 39, 41]. Very few in our study, reported ever receiving treatment for an STI. Most women with an STI infection do not experience any symptoms and consequently most infections would remain untreated [42]. Furthermore, there is increasing evidence that the “youth unfriendliness” of the primary health care facilities in South Africa may be a contributing factor to why young women do not seek help [43].

Genital lesions (sandy patches) caused by *S. haematobium* were found in almost one fifth of the young women. We found that the risk of having FGS was higher in those who had taken tablets to treat schistosomiasis previously. This may suggest that treatment did not work or that the lesions had already become chronic at the time when treatment was taken [44]. The findings suggest that the anti-schistosomal interventions have been insufficient and that reinfection has been common. Unfortunately, in South Africa, despite having high *S. haematobium* prevalence, there is no current national schistosomiasis control programme.

More than a third of the study participants had experienced multiple adverse reproductive health outcomes. Of particular public health importance, is the co-occurrence of pregnancy and STIs or HIV, which affected 21.2% of our study population. This combination may represent a particularly harmful synergy, putting both the young women and their children at increased risk for perinatal and postnatal morbidity and mortality [45]. The South African antenatal HIV prevalence survey from 2015 found that in KZN, there was an increase in HIV prevalence among pregnant women (age 15–49 years) from 37.4% in 2011 to 44.4% in 2015 [9], which corresponds well with our findings (Fig. 2). Unfortunately, the survey did not include data on STIs.

More than half of the young women had tasted alcohol but as few as 4% admitted that they had used illicit drugs. Previous studies have found substance use among young people, especially alcohol and illicit drugs to influence what they do sexually, and it is thought to place them at increased risk of practicing unsafe sex [46]. However, in our study, the number reporting alcohol and substance abuse was too small to explore these as risk factors.

Distressingly, in this study we found that young women who had sexual debut before the age of 16 years had almost four-fold higher odds of having been pregnant than those who debuted later. These adverse reproductive health outcomes can be prevented by early initiation of educational programs on puberty, sex and sexual risk behaviours prior to sexual debut (e.g. promote contraceptive use) [47]. Furthermore, our results clearly show that there is a lack of interaction between the young women and health clinics (e.g. low number of people used contraception, few had ever been tested for HIV). Making youth-friendly health services easily accessible to young people is an important factor to improve the health of young women by enabling free access to testing, treatment and contraception [43, 47, 48].

These results are not representative of the wider population, as these were school-attending, sexually active, young women. Furthermore, this is a cross sectional study which limits conclusions about the causality of the associations. Participants were tested for HIV, some STIs and FGS. However, the social and risk behaviour were self-reported and could not be confirmed independently [49]. Human papillomavirus and herpes simplex were only tested in a sub-sample and were therefore not included in this analysis even though they are important STIs. The difficulty of diagnosing FGS is a well-recognized problem, as sandy patches can be found anywhere in the genital tract as well as in the sub-mucosa [16]. Consequently, we may have under-estimated the FGS prevalence.

## Conclusion

These results confirm that there is a high prevalence of pregnancy, HIV, STIs and FGS among sexually active, school-attending young women living in rural KwaZulu-Natal, South Africa. Factors such as early sexual debut, multiple partners and older partners remained a significant risk factor for adverse reproductive health outcomes in young women. There is also an alarming co-occurrence of pregnancy and STIs or HIV. Furthermore, national school mass drug treatment programmes should be implemented to offer regular treatment to schools in schistosomiasis-endemic areas. Educational programs should be implemented to inform about safer sexual practices prior to sexual debut. Youth friendly health care facilities should be made more easily accessible for young women to obtain effective diagnosis, treatment and care.

## Abbreviations

FGS: Female genital schistosomiasis
HIV: Human immunodeficiency virus
KZN: KwaZulu-Natal
PCR: Polymerase chain reaction
STI: Sexually transmitted infections
